# Prevalence and Correlates of Truancy among School-Going Adolescents in Mozambique: Evidence from the 2015 Global School-Based Health Survey

**DOI:** 10.1155/2019/9863890

**Published:** 2019-05-23

**Authors:** Abdul-Aziz Seidu

**Affiliations:** Department of Population and Health, University of Cape Coast, Cape Coast, Ghana

## Abstract

This study examined the prevalence of truancy and its associated factors among 1500 school-going adolescents using the 2015 Mozambique Global School-based Student Health Survey data. The association was assessed using bivariate (Chi square) and multivariate (logistic regression) analysis. The prevalence of truancy was 36.6% (38.4% of males and 35.1% of females). It was found that adolescents aged 15 years and older [OR=1.460,95% CI=1.153,1.848], experiencing hunger [OR=1.613 95% CI= 1.051,2.475], current tobacco use [OR=1.613 95%CI=1.051,2.475], being bullied [OR=1.314, 95% CI=1.027,1.681], facing an attack, smoking [OR= 1.893, 95% CI=1.293,2.771], having 1-2 close friends [OR=1.656, 95% CI=1.276,2.14], and feeling lonely [OR=1.295, 95% CI=1.019,1.646] were the factors that predisposed adolescents to truant behaviour. Conversely, parental supervision [OR=0.53, 95% CI=0.232,0.791] was a protective factor against truancy. There is the need to design school-based interventions aimed at reducing truancy in Mozambique by tackling the predisposing factors and encouraging the protective factors.

## 1. Introduction

Some studies have been carried out in public health and psychology on truancy and its predictors from the high-income countries but there is scarcity of literature on truancy in the low and middle-income countries [1]. Epstein and Sheldon [2] defined truancy as students distancing themselves from school. Truancy has been regarded as both a health and social problem due to the fact that its consequences span across reproductive and sexual health, mental health, pulmonary health, injury experience, adult underachievement, and poverty [1–6].

Henry and Huizinga [7] argued that the unsupervised and unmonitored time with peers may facilitate exposure to potentially harmful lifestyles such as illicit drug use, risky sexual behaviours, and engagement in criminal activities. In Nigeria, Olley [8] found from his study that 46% of young people who lack parental supervision reported history of truancy. Formal education is a catalyst in promoting health and social well-being [1]. That notwithstanding, the attainment of education and its importance can be disturbed due to truant behaviour. Although some studies have been carried out on the prevalence of truancy among adolescents in some African countries such as Zambia [1], Swaziland, and Nigeria [9] none has been conducted in Mozambique. This study, therefore, sought to estimate the prevalence and associated factors of school truancy among adolescents in Mozambique. The results from this study can be vital in the designing of programs that can reduce or curtail the problem of truancy in Mozambique.

## 2. Methods

### 2.1. Description of the Survey and Sampling of Students

This study was cross-sectional and used data from the 2015 Global School-based Health Survey (GSHS) collected using a clustered sample design. The Mozambique GSHS was conducted in 2015. It was carried out by the World Health Organization (WHO) in collaboration with UNICEF, UNESCO, and UNAIDS with technical assistance from the Centres for Diseases Control and Prevention (CDC), Atlanta, Georgia, United States [10]. The GSHS aims to provide data on health and social behaviours among in-school adolescents. The Mozambique GSHS was a school-based survey of students in Class 8-12, which are typically attended by students aged 13-17. A two-stage cluster sample design was used to produce data representative of all students in Class 8-12 in Mozambique. The initial stage of the sampling was characterized by the selection of schools with probability proportional to enrollment size. This was followed by randomly selecting classes and all students in selected classes were eligible to participate in the study and this formed the sample size. The Mozambique GSHS measured alcohol use; dietary behaviours; drug use; hygiene; mental health; physical activity; protective factors; sexual behaviours; tobacco use; violence and unintentional injury. The students answered the survey questionnaires on a computer scannable answer sheet. The school response rate was 97%, the student response rate was 83%, and the overall response rate was 80%. A total of 1,918 students participated in the survey. However, in this study only students (1500) with complete cases on the variables that were used for the estimation were considered. Prior to commencement of the survey, permission to carry out the study was obtained from the Ministries of Health and Education. Informed consent to participate in the study was obtained from school managers and students. Students anonymously and voluntarily completed the questionnaire. The dataset is freely available for download at https://www.who.int/ncds/surveillance/gshs/mozambiquedataset/en/.

### 2.2. Variables for the Study

Students were asked: During the past 30 days, on how many days did you miss classes or school without permission? The responses were 0 days, 1 or 2 days, 3-5 days, 6-9 days, and 10 or more days. The frequency distribution of the responses to this question is 951(63.4%) which indicated that they have never absented themselves from school, 391(26.1%) indicated that they have absented themselves from school 1 to 2 days, 133 (8.9%), 11 (0.7%), and 14 (0.9%), respectively, indicated that they absented themselves from school 3 to 5 days, 6 to 9 days, and 10 or more days, respectively. The responses were transformed to a binary variable where a response of 0 was recoded as having never missed school while any number of days > 0 was recoded as 1. The independent variables were chosen from the GSHS dataset and the previous studies that have drawn conclusions on them as having association with truancy [1, 11–13].  Table 1 shows the questions and response categories and how they were recoded.

### 2.3. Data Analysis

Data analysis was performed using SATA version 14.2 software. The outcome variable was history of truancy. Due to the nature of the study design a weighting factor was used in the analysis to reflect the likelihood of sampling each pupil and to reduce bias by compensating for differing patterns of nonresponse. The analysis was done using crosstabulation to generate frequencies to describe the sample and estimate the prevalence of truancy across the various predictors. Chi square test was added to see whether the differences in proportions were significant or not. After this was done, logistic regression modelling was employed to estimate the associations between relevant predictor variables and truancy within the last 30 days. Three models were built. The first model comprised the sociodemographic variables that appeared significant in the chi square test. The second model added tobacco use, fighting, smoking, bullying, number of close friends, and suicidal ideation. The last model added parental checking of homework to see how all the variables were associated with truancy (see Table 3). In terms of the usefulness of the models, model 1 as a whole explained 1% (Pseudo R^2^) of the variance in truancy, model II explained about 9% (Pseudo R^2^) of the variance in truancy, and model III also explained about 9% (Pseudo R^2^) of the variance in truancy. The results were reported as odds ratios (OR) together with their 95% confidence intervals (CI) for selected predictor variables while considering having been truant in the last 30 days as a dependent variable.

## 3. Results

A total of 1500 pupils (46.2% males and 53.8% females) were used for the study. Twenty-five percent each were in 9^th^ and 10^th^ classes while 64.1% were aged 15 years and above. Overall 36.6% of the participants (38.4%% of males and 35.1% of females) reported being truant in the past 30 days before the day of the data collection. The chi square test showed statistically significant differences in the associations between age (X^2^=11.17, p<0.001), experiencing hunger (X^2^=14.15, p<0.001), smoking (X^2^=85.27, p<0.001), tobacco use (X^2^=77.33, p<0.001), engaging in fight (X^2^=21.20, p<0.001), bullying victimization (X^2^=47.91, p<0.001), suicidal ideation (X^2^=24.43, p<0.001), number of close friends (X^2^=21.72, p<0.001), parents checking homework (X^2^=7.49, p<0.010), and truancy among adolescents in Mozambique (see Table 2).

### 3.1. Multivariate Analysis on the Association between Independent Variables and Truancy

As shown in Table 3, when all the confounding variables were controlled for, the multivariate analysis revealed that age, hunger, smoking, bullying, number of close friends, loneliness, and parents checking homework were associated with truancy. Specifically, adolescents aged 15 years and above were more likely to be truants compared to those aged 11-14 years [OR=1.460,95% CI=1.153,1.848]. The adolescents who also indicated that they experienced hunger [OR=1.613, 95% CI= 1.051,2.475] were about 1.6 times more likely to be truants compared to those who did not experience hunger. Those who used tobacco were more likely to be truant compared to those who did not use tobacco [1.613, 95% CI= 1.051,2.475]. Smoking was also significantly associated with truancy among adolescents in Mozambique. The adolescents who smoked were about 1.8 more likely to be truants compared to those who did not smoke [OR= 1.893,95%CI=1.293,2.771]. Adolescents who were also bullied [OR=1.314, 95% CI=1.027,1.681], those with 1-2 number of close friends [OR=1.656, 95% CI=1.276,2.14], and those who felt lonely [OR=1.295, 95% CI=1.019,1.646] were more likely to be truants compared to those who were not bullied, those who did not have close friends, and those who did not feel lonely, respectively. The adolescents whose parents checked their homework [OR=0.53, 95% CI=0.232,0.791] were less likely to be truants compared to those whose parents or guardians did not check their homework (see Table 3).

## 4. Discussion

It was found that the prevalence of truancy is 36.6% among the adolescents in Mozambique. Although both the bivariate and the multivariate analysis did not show statistically significant relationship between the sex of the respondents and truancy, the proportions of males who were truants were 38.4% and 35.1% were females. There is about 3% difference in terms of the sexes. The higher proportion of males being truants compared to females could be what Siziya et al. [11] explained as … “a manifestation of cultural expectations and that truancy among boys may be more tolerated than truancy among girls”. The overall proportion of 36.6% of truancy among adolescents in Mozambique is similar to what was found in Malaysia by Hidayah et al. [14]; Shah et al. [15]; and Yoep et al. [13] as well as what WHO [16] found in Indonesia. Nonetheless, it was lower than what was found in Myanmar [12], Thailand [17], Ghana [18], and Swaziland [11] but significantly lower than Zambia [1].

It was also found that adolescents aged 15 years and above were more likely to be truants compared to those aged 11-14 years. Similar results were found by Muula et al. [1] in Zambia. They found that adolescents in the younger age category–14 years and below were less likely to be truants compared to older adolescents. They explained that younger students are more likely to be under parental supervision than older pupils and may thus be less likely to be truants than older pupils. The adolescents who also indicated that they experienced hunger were about 1.6 times more likely to be truants compared to those who did not experience any hunger. The results confirm what was found in previous studies [1, 11, 12, 18]. Muula et al. [1] and Seidu et al. [18] explained that most students who feel hungry are from poor households and as a result might absent themselves from school because of work engagement at home.

As evidenced in previous studies [1, 11, 15, 18] adolescents who smoked and used tobacco were more likely to be truants. The explanation offered by Peltzer and Pengpid [12] is that truanting adolescents might have more unsupervised time in order to engage in tobacco and alcohol use. Relatedly, it was found that study participants who reported being victims of bullying were more likely to be truants. Seidu et al. [18] and Peltzer and Pengpid [12] explained that adolescents who have experienced bullying victimization may miss school in order to escape further victimization by their peers. It is therefore important that, to ensure a reduction in truancy, a bully-free school is needed [1, 19]. In this current study, those with 1-2 number of close friends and those who felt lonely were more likely to be truants. The results on the number of close friends and its relationship with truancy are consistent with previous studies by Muula et al. [1] and Henry and Huizinga [7]. They found that adolescents with delinquent peers were a strong predictor of truancy. It is possible that truant students with their colleague truant students might be engaging in similar behaviours.

The adolescents whose parents checked their homework were less likely to be truants compared to those whose parents or guardians did not check their homework. This resonates what was found in previous studies [1, 11, 12, 20]. They indicated that pupils who reported that their parents or guardians checked their homework or reported that they received parental supervision were less likely to be truants. Despite this, a study by Miller and Plant [21] did not show parental caring and control as protective against truancy.

The strength of this study is based on the fact that it utilized data from a large nationally representative survey with a relatively large sample size. The study, however, has some limitations that need to be acknowledged. Given the cross-sectional nature of the data used, inferences between the independent variables of the adolescents and truancy could only be based on associations rather than causal links. The students also filled the survey questionnaire themselves; due to this, there is the possibility of social desirability responses. In addition, the specific outcome that the study sought to measure, thus truancy, and the students who were absent on the day of the data collection were exempted and this might lead to under reporting of the phenomenon. The grouping of the outcome variable to a dichotomous variable could not give a deeper insight on the factors associated with those who just absented themselves from school for few (1-5) days and those who were absent for more than 5 days. Excluding the missing cases from the study might have led to a reduction in the sample size nonetheless; this did not reduce the statistical power as the sample size was relatively large. Finally, the study excluded out-of-school adolescents.

## 5. Conclusion

It is concluded that the prevalence of school truancy within the past 30 days among in-school adolescents in Mozambique was 36.6% which is relatively high. Experiencing hunger, current tobacco use, being bullied, facing an attack, smoking, having 1-2 close friends, and feeling lonely were the factors that predisposed adolescents to truant behaviour. In contrast, parental supervision was a protective factor against truancy. There is the need to design school-based interventions aimed at reducing truancy in Mozambique by tackling the predisposing factors and encouraging the protective factors.

## Figures and Tables

**Table 1:** Variable description.

Variables	Question	Response options and recoding
Outcome variable		

Truancy	During the past 30 days, on how many days did you miss classes or school without permission?	1 = 0 days, 2 = 1 or 2 days, 3 = 3 to 5 days, 4 = 6 to 9 days, 5 = 10 or more *(coded 1 = 0 and 2-5 = 1)*

Independent variables		
Age	Custom age	1 = 12, 2 = 13, 3 = 14, 4 = 15, 5 = 16, 6 = 17,7 = 18 years *(Coded as 1 = 12-14 and 2 = 15+)*
Sex	Sex	1 = male, 2 = female
Grade	In what grade are you?	1 = 8 to, 5 = 12
		
Hunger	During the past 30 days, how often did you go hungry because there was not enough food in your home?	1 = never, 2 = Rarely, 3 = sometimes, 4 = most of the times, 5 = always *(coded 1 = 0 and 2-5 = 1)*
Tobacco use	“During the past 30 days, on how many days did you use any other form of tobacco, such as chewing tobacco leaves?”	1* * = * *0 days; to 7* * = * *All 30 days *(coded as 1 = 0; and 2–7 = 1)*
Smoking	During the past 30 days, on how many days did you smoke cigarettes?	1* * = * *0 days; to 7* * = * *All 30 days *(coded as 1 = 0; and 2–7 = 1)*
Fight	During the past 12 months, how many times were you in a physical fight?	1* * = * *0 times; to 8* * = * *12 or more times *(coded 1 = 0; and 2–8 = 1)*
Bullied	During the past 30 days, how were you bullied most often?	1* * = * *0 times; to 8* * = * *12 or more times *(coded 1 = 0; and 2–7 = 1)*
Attack	During the past 12 months, how many times were you physically attacked?	1* * = * *0 times; to 8* * = * *12 or more time *(coded as 1 = 0; and 2–8 = 1)*
Close friends	How many close friends do you have?”	1 = 0 to 4 = 3 or more *(coded as 1 = 0, 1-2 = 1 and 3 or more = 2)*
Feeling Lonely	During the past 12 months, how often have you felt lonely?	1* * = * *never; to 5* * = * *always *(coded 1 = 0; and 2–5 = 1)*
Suicidal ideation	During the past 12 months, did you ever seriously consider attempting suicide?”	1* * = * *yes, 2* * = * *no * (coded 2 = 0; and 1 = 1)*
		
Helpful (Peer support)	During the past 30 days, how often were most of the students in your school kind and helpful?	1* * = * *never; to 5* * = * *always *(coded 1 = 0; and 2–5 = 1)*
Parents check homework (parental supervision)	During the past 30 days, how often did your parents or guardians check to see if your homework was done?	1* * = * *never; to 5* * = * *always *(coded 1 = 0; and 2–5 = 1)*
Understand problems (Parental connectedness	During the past 30 days, how often did your parents or guardians understand your problems and worries?	1* * = * *never; to 5* * = * *always *(coded 1 = 0; and 2–5 = 1)*
Know what adolescent do free time (Parental or guardian Bonding)	During the past 30 days, how often did your parents or guardians really know what you were doing with your free time?”	1* * = * *never; to 5* * = always *(coded 1 = 0; and 2–5 = 1)*

**Table 2:** Chi-square analysis on the proportions of truancy among in-school adolescents in Mozambique.

Variables	Truancy (n=1500)		Chi-square (X^2^)	P-Value
	No	Yes		
	63.4	36.6		
Socio-demographic factors				
*Age*			11.17	p<0.001*∗∗∗*
11-14yrs	69.0	31.0		
15+	60.3	39.7		
*Sex*			1.77	0.184
Male	61.6	38.4		
Female	64.9	35.1		
*Grade*			1.10	0.776
8^th^ and 9^th^	63.8	36.2		
10^th^	65.3	34.7		
11^th^	62.0	38.0		
12^th^	62.1	37.9		
*Experienced Hunger*			14.15	p<0.001*∗∗∗*
No	69.0	31.0		
Yes	59.5	40.5		
*Tobacco use *			77.33	p<0.001*∗∗∗*
No	67.7	32.3		
Yes	35.4	64.7		
Smoking				p<0.001*∗∗∗*
No	68.4	31.6	85.28	
Yes	37.1	62.9		
*Engaged in a fight*			28.20	p<0.001*∗∗∗*
No	70.6	29.4		
Yes	57.4	42.7		
*Bullied*			47.91	p<0.000*∗∗∗*
No	70.9	29.1		
Yes	53.5	46.5		
*Attacked *			62.14	p<0.001*∗∗∗*
No	73.1	26.9		
Yes	53.5	46.6		
*Suicidal ideation*			24.43	p<0.001*∗∗∗*
No	66.0	34.0		
Yes	48.7	51.4		
*Number of Close friends*			21.72	p<0.001*∗∗∗*
*0*	71.6	28.4		
1-2	55.9	44.1		
3 or more	67.5	32.5		
*Felt lonely*			21.15	p<0.001*∗∗∗*
No	70.9	29.1		
Yes	59.0	41.0		
*Peer support*				
No	66.7	33.3	1.62	0.203
Yes	62.6	37.4		
*Parents checking homework *			7.49	p<0.01*∗∗*
No	58.7	41.3		
Yes	65.9	34.1		
*Parents understood problems*			3.81	0.051
No	61.0	39.0		
Yes	65.9	34.1		
*Parents check at free Time*			0.33	0.564
No	62.6	37.4		
Yes	64.1	35.9		

**Table 3:** Multivariate analysis on the association between independent variables and truancy.

Variable	Model I OR (95% CI)	Model II OR (95% CI)	Model III OR (95% CI)
*Age *			
11-14	Ref	Ref	Ref
15+	1.463*∗∗∗*[1.168,1.833]	1.475*∗∗∗*[1.165,1.868]	1.460*∗∗*[1.153,1.848]
*Experienced hunger*			
No	Ref	Ref	Ref
Yes	1.515*∗∗∗*[1.218,1.884]	1.239[0.983,1.561]	1.229[0.975,1.550]
*Tobacco use *			
No	–	Ref	Ref
Yes	–	1.599*∗*[1.045,2.446]	1.613*∗*[1.051,2.475]
*Engaged in a fight*			
No	–	Ref	Ref
Yes	–	1.083 [0.835,1.404]	1.071 [0.825,1.389]
*Smoking*			
No	–	Ref	Ref
Yes	–	1.884*∗∗*[1.289,2.754]	1.893*∗∗*[1.293,2.771]
*Bullied *			
No	–	Ref	Ref
Yes	–	1.341*∗*[1.050,1.713]	1.314*∗*[1.027,1.681]
*Attacked *			
No	–	Ref	Ref
Yes	–	1.662*∗∗∗*[1.282,2.156]	1.656*∗∗∗*[1.276,2.14]
*Number of Close friends*			
0	–	Ref	Ref
1-2	–	2.046*∗*[1.163,3.600]	2.131*∗*[1.214,3.740]
3+	–	1.335 [0.764,2.331]	1.382 [0.793,2.407]
*Felt Lonely *			
No	–	Ref	Ref
Yes	–	1.283*∗*[1.010,1.630]	1.295*∗*[1.019,1.646]
*Suicidal Ideation *			
No	–	Ref	Ref
Yes	–	1.310[0.933,1.841]	1.288[0.914,1.815]
*Parents checking Homework *			
No	–	Ref	Ref
Yes			0.53*∗*[0.232,0.791]

N	1500	1500	1500

Pseudo R^2^	0.013	0.089	0.091

*∗* p<0.05, *∗∗* p<0.01, and *∗∗∗* p<0.001; CI=confidence interval; Ref=reference; OR=odds ratio.

## Data Availability

The data set is freely available for download at https://www.who.int/ncds/surveillance/gshs/datasets/en/.
